# Body weight and BMI variability linked to dementia risk: A meta-analysis

**DOI:** 10.17305/bb.2025.12626

**Published:** 2025-07-07

**Authors:** Sitian Fang, Lewei Guan, Huimin Jian, Xi-jian Dai, Lianggeng Gong

**Affiliations:** 1 Department of Radiology, The Second Affiliated Hospital, Jiangxi Medical College, Nanchang University, Nanchang 330006, China; 2 Intelligent Medical Imaging of Jiangxi Key Laboratory, Nanchang 330006, China; 3 Huankui Academy, Nanchang University, Nanchang 330031, China; 4 The Second Clinical Medical College, Nanchang University, Nanchang 330031, China

**Keywords:** Body weight, BW, variability, dementia, Alzheimer’s disease, AD, risk factor

## Abstract

Emerging evidence suggests that fluctuations in body weight (BW) or body mass index (BMI), independent of average levels, may influence dementia risk. However, the association between intra-individual variability in BW or BMI and incident dementia remains unclear. This meta-analysis aimed to clarify this relationship. A systematic search of PubMed, Embase, and Web of Science was conducted through March 25, 2025, to identify longitudinal observational studies reporting dementia outcomes in relation to BW or BMI variability. Relative risks (RRs) comparing the highest versus lowest variability categories were pooled using a random-effects model. Subgroup and sensitivity analyses were performed to explore heterogeneity and assess the robustness of the results. Nine cohort studies (10 datasets; 4,232,666 participants) were included. Overall, high BW or BMI variability was associated with a significantly increased risk of dementia (RR ═ 1.36, 95% CI: 1.27–1.46; *P* < 0.001; *I^2^* ═ 84%). The association was consistent for both BW (RR ═ 1.45) and BMI (RR ═ 1.34) variability. Subgroup analyses showed stronger associations in prospective studies than in retrospective ones, and in studies that did not adjust for baseline BW/BMI compared to those that did (*P* for subgroup difference < 0.05). Associations remained robust in sensitivity analyses and across dementia subtypes, including Alzheimer’s disease and vascular dementia. No significant publication bias was detected (Egger’s test, *P* ═ 0.22). In conclusion, greater intra-individual variability in BW or BMI may be independently associated with increased dementia risk. These findings underscore the importance of maintaining weight stability in mid-to-late life as a potential preventive strategy for dementia.

## Introduction

Dementia is a progressive neurodegenerative syndrome characterized by cognitive decline, functional impairment, and loss of independence [1, 2]. Currently, over 55 million individuals worldwide are living with dementia, a figure projected to triple by 2050 due to population aging. Alzheimer’s disease (AD) and vascular dementia (VD) are the two most prevalent subtypes, collectively accounting for the majority of cases [3–5]. Despite advancements in symptomatic treatments and recent efforts toward disease-modifying therapies, dementia remains incurable, imposing a significant burden on patients, families, and healthcare systems [5]. Given the limited effectiveness of existing treatments, identifying modifiable risk factors for early prevention and intervention has become a major public health priority [6, 7]. Established risk factors for dementia include age, genetics (e.g., APOE ɛ4), cardiovascular disease, and lifestyle factors; however, many cases remain unexplained, highlighting the need to investigate novel predictors [8].

In recent years, increasing attention has been directed toward the role of intra-individual variability in body composition—body weight (BW) and body mass index (BMI)—as potential indicators of health instability [9]. Variability in BW or BMI is typically quantified using statistical metrics such as standard deviation (SD), coefficient of variation (CV), average successive variability (ASV), or variability independent of the mean (VIM), derived from serial measurements over time [10]. Unlike static values of BW or BMI, which are well-documented in their associations with various chronic diseases, variability reflects dynamic physiological and behavioral changes [9, 10]. Previous studies have linked fluctuations in BW or BMI to increased risks of mortality, cardiovascular events, and metabolic disturbances, potentially mediated by mechanisms such as chronic inflammation, autonomic dysregulation, and impaired homeostasis [11–13].

The potential relationship between BW/BMI variability and cognitive dysfunction or dementia has recently emerged as a focal point of research [14]. Fluctuations in BW may indicate underlying frailty, neuroendocrine disruption, or nutritional instability—all factors implicated in cognitive decline [15, 16]. However, existing studies on this topic have yielded inconsistent findings, and the strength and direction of the association remain unclear [17–25]. Furthermore, variability in measurement methods, study populations, and dementia outcomes has contributed to heterogeneity in results. Therefore, this study aims to perform a meta-analysis to systematically evaluate the association between intra-individual variability in BW or BMI and the risk of incident dementia.

## Materials and methods

This meta-analysis was conducted in accordance with the PRISMA 2020 statement [26, 27] and the Cochrane Handbook for Systematic Reviews [28], which guided the development of the protocol, data collection, statistical synthesis, and reporting. The protocol has been prospectively registered in the PROSPERO database under the identifier CRD420251043561.

### Database search

To identify studies relevant to this meta-analysis, we searched PubMed, Embase, and Web of Science databases using an extensive array of search terms, including: (1) “body weight” OR “body mass index” OR “BMI”; (2) “variation” OR “variability” OR “fluctuation” OR “oscillation” OR “fluctuate”; (3) “dementia” OR “Alzheimer” OR “Alzheimer’s” OR “cognitive decline” OR “cognitive impairment” OR “cognitive dysfunction” OR “cognition”; and (4) “prospective” OR “prospectively” OR “longitudinal” OR “incident” OR “incidence” OR “risk” OR “followed” OR “follow-up” OR “cohort”. The literature search was limited to studies involving human participants and included only full-length, peer-reviewed articles published in English. To ensure comprehensive coverage, the reference lists of relevant original and review articles were also manually screened for additional eligible studies. The search spanned from the inception of each database through March 25, 2025, with detailed search strategies provided in Supplemental File 1.

### Study selection

The inclusion criteria were structured according to the PICOS framework:

*Population (P):* Adults aged 18 years or older without dementia at baseline.

*Exposure (I):* High intra-individual variability in BW or BMI, as defined by original studies employing quantitative measures. Exposure classification adhered to the original cutoffs established in each study.

*Comparison (C):* Individuals exhibiting low BW or BMI variability at baseline.

*Outcome (O):* Incident cases of all-cause dementia, AD, or VD during follow-up, with diagnostic definitions and validation methods consistent with those utilized in the respective studies.

*Study design (S):* Longitudinal observational studies, encompassing cohort studies, nested case-control designs, and post-hoc analyses of clinical trials.

Exclusion criteria comprised reviews, editorials, meta-analyses, preclinical studies, and studies that included participants with dementia at baseline, lacked a defined measure of BW or BMI variability, or did not report incident dementia outcomes. In instances of overlapping populations, the study with the largest and most comprehensive dataset was selected for inclusion.

### Study quality evaluation and data collection

The literature search, study selection, quality assessment, and data extraction were conducted independently by two reviewers, with any disagreements resolved through discussion with the corresponding author. Study quality was assessed using the Newcastle–Ottawa Scale (NOS), which evaluates three domains: participant selection, control for confounding, and outcome assessment [29]. The NOS assigns scores ranging from 1–9, with higher scores indicating superior quality; studies scoring 7 or above were classified as high quality. Extracted data encompassed study-level information (first author, publication year, country, and study design), participant characteristics (source population, number of subjects, mean age, and sex), methods of measuring BW or BMI variability (number and timing of measurements, variability metrics, and cutoffs used), follow-up duration, dementia diagnosis methods, types of dementia outcomes reported (all-cause, AD, or VD), number of incident dementia cases, and covariates adjusted for in the association analyses.

### Statistical analysis

The association between BW or BMI variability and the risk of dementia was assessed by pooling relative risks (RRs) and their corresponding 95% confidence intervals (CIs). This analysis compared individuals in the highest and lowest categories of BW/BMI variability at baseline. The estimates were directly extracted from the original studies without converting continuous measures (e.g., per-standard deviation hazard ratios) into categorical contrasts. Consequently, no transformation methods were applied. When necessary, RRs and their standard errors were calculated from reported 95% CIs or *P* values, followed by log transformation to stabilize variance and normalize the distribution [28].

Between-study heterogeneity was evaluated using the Cochrane *Q* test and the *I*^2^ statistic, with thresholds of < 25%, 25%–75%, and > 75% interpreted as low, moderate, and high heterogeneity, respectively [30]. A random-effects model was employed to accommodate expected variation across studies [31]. Additionally, sensitivity analyses were conducted using the Hartung–Knapp–Sidik–Jonkman (HKSJ) method with restricted maximum likelihood (REML) estimation to assess the robustness of the results under a more conservative variance estimator. Sensitivity analyses also involved sequentially omitting each study to evaluate the stability of the pooled estimates.

**Figure 1.**
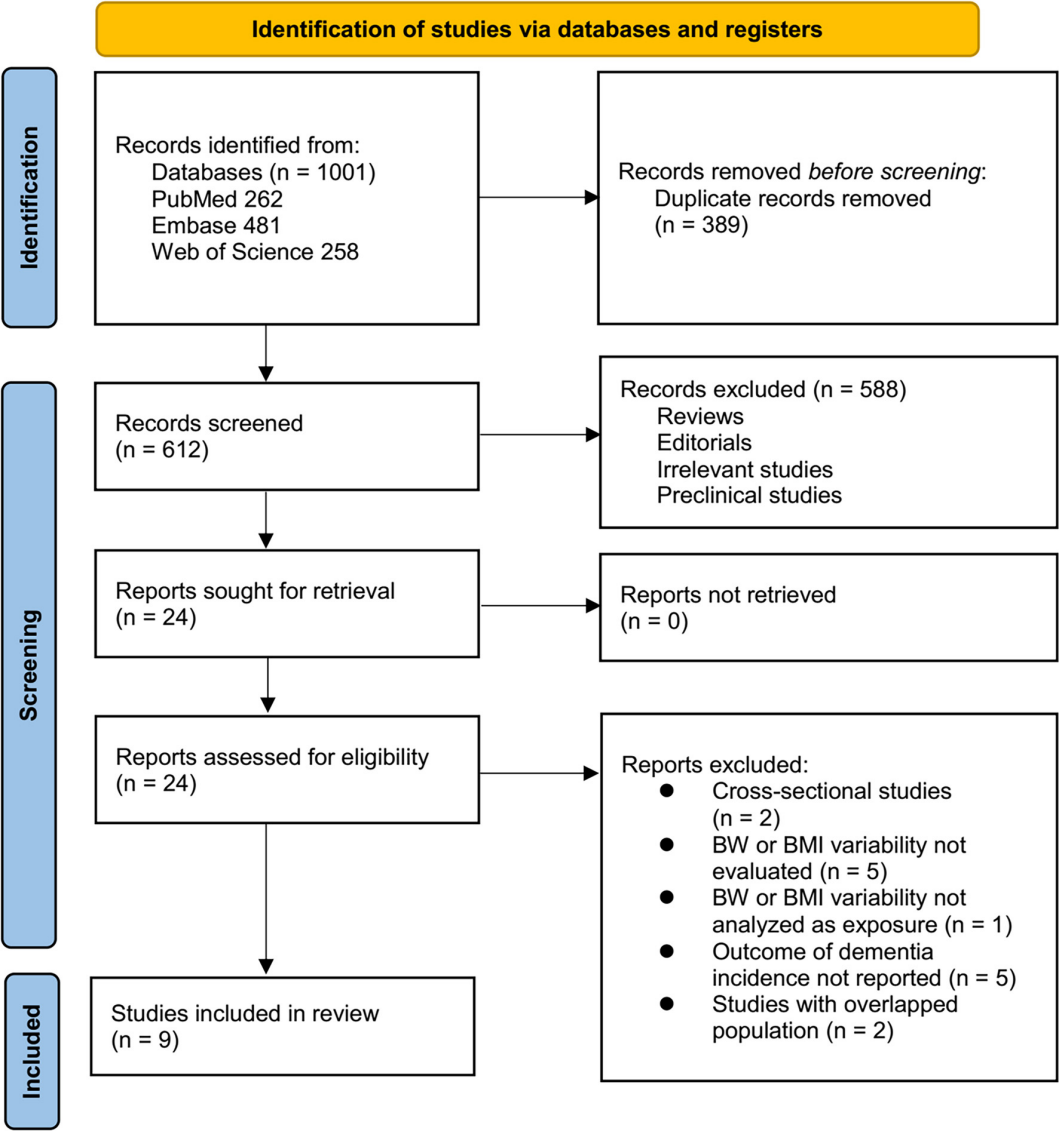
**Flow diagram of study selection.** A total of 1001 records were initially identified through database searches and citation screening. After removing 389 duplicates, 612 records were screened based on title and abstract, of which 588 were excluded for not meeting the eligibility criteria. The full texts of the remaining 24 articles were reviewed in detail, resulting in the exclusion of 15 studies for reasons specified in the diagram. Ultimately, nine studies were included in the final quantitative synthesis.

Subgroup analyses were performed to explore the influence of study-level characteristics, including the type of variability assessed (BW vs BMI), dementia outcomes (AD or VD), study design (prospective vs retrospective), baseline age group (≥ 60 years vs ≥ 40 years), proportion of male participants, follow-up duration, method of dementia validation (clinical diagnosis, ICD coding, or self/proxy report), and whether baseline BW/BMI was adjusted for in the analysis. Median values of continuous variables were used to establish subgroup cutoffs. Publication bias was assessed through visual inspection of funnel plots and formally tested using Egger’s regression test [6]. Atest, with a *P* value < 0.05 indicating statistical significance. All statistical analyses were conducted using RevMan (version 5.1; Cochrane Collaboration, Oxford, UK) and Stata (version 12.0; Stata Corporation, College Station, TX, USA).

**Table 1:** Characteristics of the included studies

**Study**	**Country**	**Study design**	**Population characteristics**	**No. of subjects**	**Mean age (years)**	**Men (%)**	**Times and durations for determining BW/BMI variability**	**Parameters for evaluating BW/BMI variability**	**Cutoff for evaluating BW/BMI variability**	**Follow-up duration (years)**	**Dementia outcome reported**	**Outcome validation**	**No. of patients with dementia**	**Variables adjusted**
Ravona et al., 2013	Israel	PC	Apparently healthy men aged 40–70 years	1620	44.3	100	BW measured at 3 time points in 5 years	SD-BW	Q4:Q1	36	Overall dementia	TICS-m telephone screening and in-person neurologist/psychiatrist assessment using DSM-IV criteria	307	Age, SES, height, DM, baseline weight, BP, cholesterol, smoking, physical activity, dietary intake, and intentional dieting
Lee et al., 2018	South Korea	PC	General population aged ≥45 years without history of hypertension, DM, or dyslipidemia	2930816	54.4	50.9	BW measured ≥3 times between 2005 and index year	CV-BMI	Q4:Q1	5.5	Overall dementia, AD, and VD	ICD-10 codes and prescription of dementia medications	32901 (AD: 24486, VD: 3629)	Age, sex, smoking, alcohol consumption, physical activity, income, baseline BMI, BP, glucose, and TC
Roh et al., 2020	South Korea	RC	Older adults aged ≥ 67 years	19987	73.1	60.2	BW measured ≥3 times in 4 years	VIM-BW	Q4:Q1	6.5	Overall dementia, AD, and VD	ICD codes with documented cognitive decline	1592 (AD: 1217, VD: 304)	Age, sex, BMI at baseline, smoking, alcohol, exercise, income, DM, hypertension, dyslipidemia, IHD, and cerebrovascular disease
Bae et al., 2021	South Korea	PC	Adults aged ≥45 years with normal cognitive function at baseline	3477	NR	53.7	BW measured at 3 time points in 4 years	ASV-BMI	Q5:Q1	10	Overall dementia	K-MMSE ≤17	NR	Age, sex, marital status, education, income, health insurance, region, smoking, alcohol, physical activity, comorbidities, depression, and BMI at baseline
Kang et al., 2021	South Korea	RC	Adults aged ≥60 years	45076	NR	51.7	BW measured at 3 time points in 5 years	ASV-BMI	Q4:Q1	2.7	AD	ICD-10 codes, prescription of anti-dementia drugs, and documentation of cognitive dysfunction	4055	Age, sex, insurance premium, BMI at baseline, smoking, alcohol, physical activity, hypertension, DM, and dyslipidemia
Park et al., 2022	South Korea	RC	Adults aged ≥40 years with T2DM and no prior dementia	1206764	59.4	62.5	BW measured ≥3 times in 5 years	VIM-BW	Q4:Q1	7.9	Overall dementia, AD, and VD	ICD-10 codes and prescription of dementia medications	162615 (AD: 65169, VD: 18705)	Age, sex, smoking, alcohol, exercise, income, hypertension, dyslipidemia, insulin use, number of oral antidiabetics, DM duration, baseline BW
Chen et al., 2022	USA	PC	Adults aged ≥70 years, cognitively intact	5547	71.1	43.3	BW measured at 9 time points in 16 years	CV-BW	Q4:Q1	6.8	Overall dementia and AD	Self/proxy report of physician-diagnosed dementia	427 (AD: 201)	Age, sex, race, education, income, smoking, alcohol, exercise, weight change over 16 years, and comorbidities
Wang et al., 2024	USA	PC	Non-demented adults aged ≥65 years	542	74.6	57	BW measured at 5 time points in 4 years	SD-BMI	Q4:Q1	8	AD	NINCDS-ADRDA clinical diagnostic criteria	285	Age, sex, education, APOE ɛ4 status, baseline cognitive status, hypertension, DM, smoking, and intracranial volume
Wu et al., 2024	Australia and USA	PC	Community-dwelling individuals aged ≥65 years, cognitively intact and free of major illness at baseline	18837	NR	44	BW measured at 3 time points in 2 years	SD-BMI	T3:T1	6.3	Overall dementia	DSM-IV criteria	844	Age, sex, education, ethnicity, living situation, smoking, alcohol, hypertension, DM, dyslipidemia, depression, pulse pressure, TG, and APOE ɛ4

PC: Prospective cohort; RC: Retrospective cohort; BW: Body weight; BMI: Body mass index; SD: Standard deviation; CV: Coefficient of variation; VIM: Variability independent of the mean; ASV: Average successive variability; Q1: First quartile; Q4: Fourth quartile; Q5: Fifth quintile; T1: First tertile; T3: Third tertile; AD: Alzheimer’s disease; VD: Vascular dementia; DSM-IV: Diagnostic and Statistical Manual of Mental Disorders, Fourth Edition; ICD-10: International Classification of Diseases, Tenth Revision; TICS-m: Modified Telephone Interview for Cognitive Status; MMSE: Mini-Mental State Examination; K-MMSE: Korean Mini-Mental State Examination; CDR: Clinical dementia rating; GDS: Global deterioration scale; NINCDS-ADRDA: National Institute of Neurological and Communicative Disorders and Stroke–Alzheimer’s Disease and Related Disorders Association; SES: Socioeconomic status; DM: Diabetes mellitus; BP: Blood pressure; TC: Total cholesterol; IHD: Ischemic heart disease; TG: Triglycerides; APOE ɛ4: Apolipoprotein E epsilon 4 allele; NR: Not reported.

## Results

### Study retrieval

The study selection process is depicted in Figure 1. Initially, 1,001 potentially relevant records were identified through database searches and citation screening. Following the removal of 389 duplicates, 612 records remained for title and abstract screening, resulting in the exclusion of 588 articles that did not align with the objectives of the meta-analysis. The full texts of the remaining 24 articles were independently assessed by two reviewers, which led to the exclusion of 15 studies for the reasons detailed in Figure 1. Ultimately, nine studies met the inclusion criteria and were incorporated into the quantitative synthesis [17–25].

### Overview of the study characteristics

Table 1 presents a summary of the characteristics of the nine studies included in this meta-analysis, published between 2013 and 2024, and conducted in Israel, South Korea, the United States, and Australia. All studies are longitudinal cohorts, comprising six prospective [17, 18, 20, 22, 24, 25] and three retrospective [19, 21, 23], encompassing a total of 4,232,666 participants. The study populations included middle-aged to older adults, with mean ages ranging from 44.3–74.6 years, and the proportion of male participants varied from 43.3% to 100%. Intra-individual variability in BW [17, 19, 22, 23] or BMI [18, 20, 21, 24, 25] was assessed using statistical parameters such as SD, CV, ASV, and VIM. The number and timing of repeated BW/BMI measurements varied from three to nine time points over periods of two to sixteen years, with comparisons typically made between the highest and lowest variability categories, such as quintiles [20], quartiles [17–19, 21–24], and tertiles [25]. All studies reported incident dementia outcomes, including all-cause dementia in seven studies [17–20, 22, 23, 25], AD in six studies [18, 19, 21–24], and VD in three studies [18, 19, 23]. Dementia diagnoses were validated using various methods, including clinical assessments [17, 20, 24, 25], ICD-10 codes accompanied by medication records or structured cognitive tests [18, 19, 21, 23], and self/proxy physician reports [22]. Follow-up durations ranged from 2.7–36 years, with outcome ascertainment aligned with each study’s protocol. Most studies adjusted for a comprehensive set of covariates, including age, sex, education, and comorbidities; however, three studies did not account for baseline BW or BMI, potentially influencing the observed associations [22, 24, 25]. As illustrated in Table 2, the NOS scores ranged from 6–9, indicating moderate to high methodological quality. Notably, three studies achieved the maximum score of 9 stars [17, 24, 25], three studies scored 8 stars [18, 20, 22], and three studies scored either 7 [19, 23] or 6 [21] stars, primarily due to limitations in follow-up duration or outcome ascertainment.

**Table 2:** Study quality evaluation via the Newcastle–Ottawa scale

**Study**	**Representativeness of the exposed cohort**	**Selection of the non-exposed cohort**	**Ascertainment of exposure**	**Outcome not present at baseline**	**Control for age and sex**	**Control for other confounding factors**	**Assessment of outcome**	**Enough long follow-up duration**	**Adequacy of follow-up of cohorts**	**Total**
Ravona et al., 2013	1	1	1	1	1	1	1	1	1	9
Lee et al., 2018	1	1	1	1	1	1	0	1	1	8
Roh et al., 2020	0	1	1	1	1	1	0	1	1	7
Bae et al., 2021	1	1	1	1	1	1	1	1	1	8
Kang et al., 2021	0	1	1	1	1	1	0	0	1	6
Park et al., 2022	0	1	1	1	1	1	0	1	1	7
Chen et al., 2022	1	1	1	1	1	1	0	1	1	8
Wang et al., 2024	1	1	1	1	1	1	1	1	1	9
Wu et al., 2024	1	1	1	1	1	1	1	1	1	9

### Association between variability of BW/BMI and dementia risk

**Figure 2.**
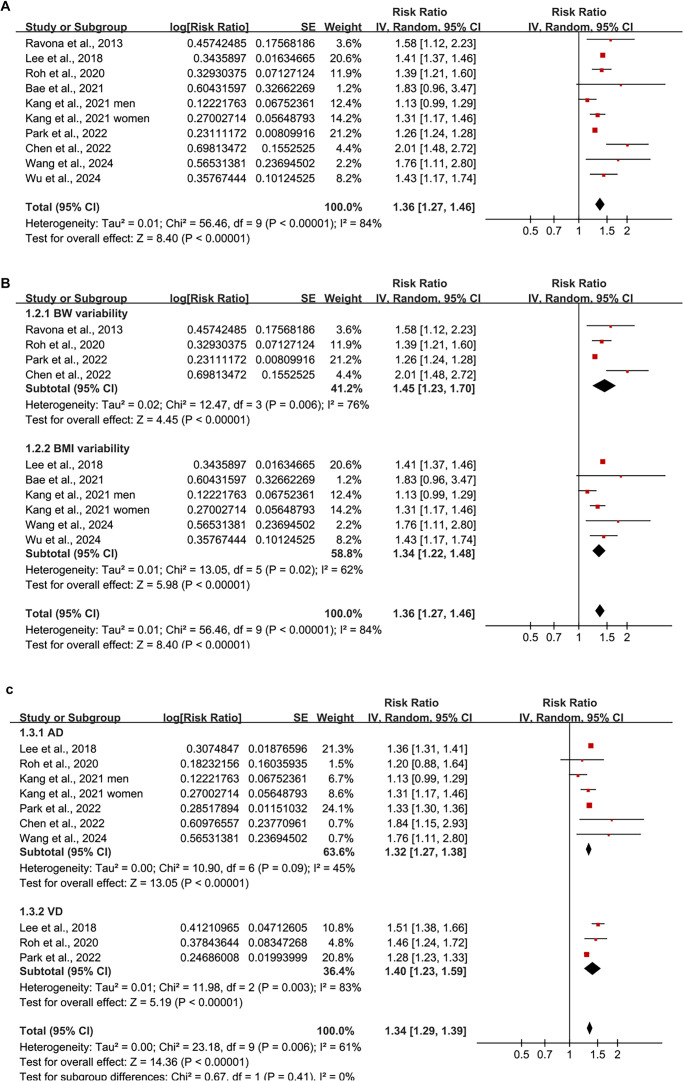
**Forest plot of the association between body weight or BMI variability and risk of dementia.** (A) Pooled analysis comparing the highest versus lowest variability categories shows that greater intra-individual variability in BW or BMI is significantly associated with increased dementia risk; (B) Subgroup analysis by type of exposure (BW vs BMI variability); (C) Subgroup analysis by type of dementia (AD vs VD). BW: Body weight; BMI: Body mass index; AD: Alzheimer’s disease; VD: Vascular dementia.

**Figure S1.**
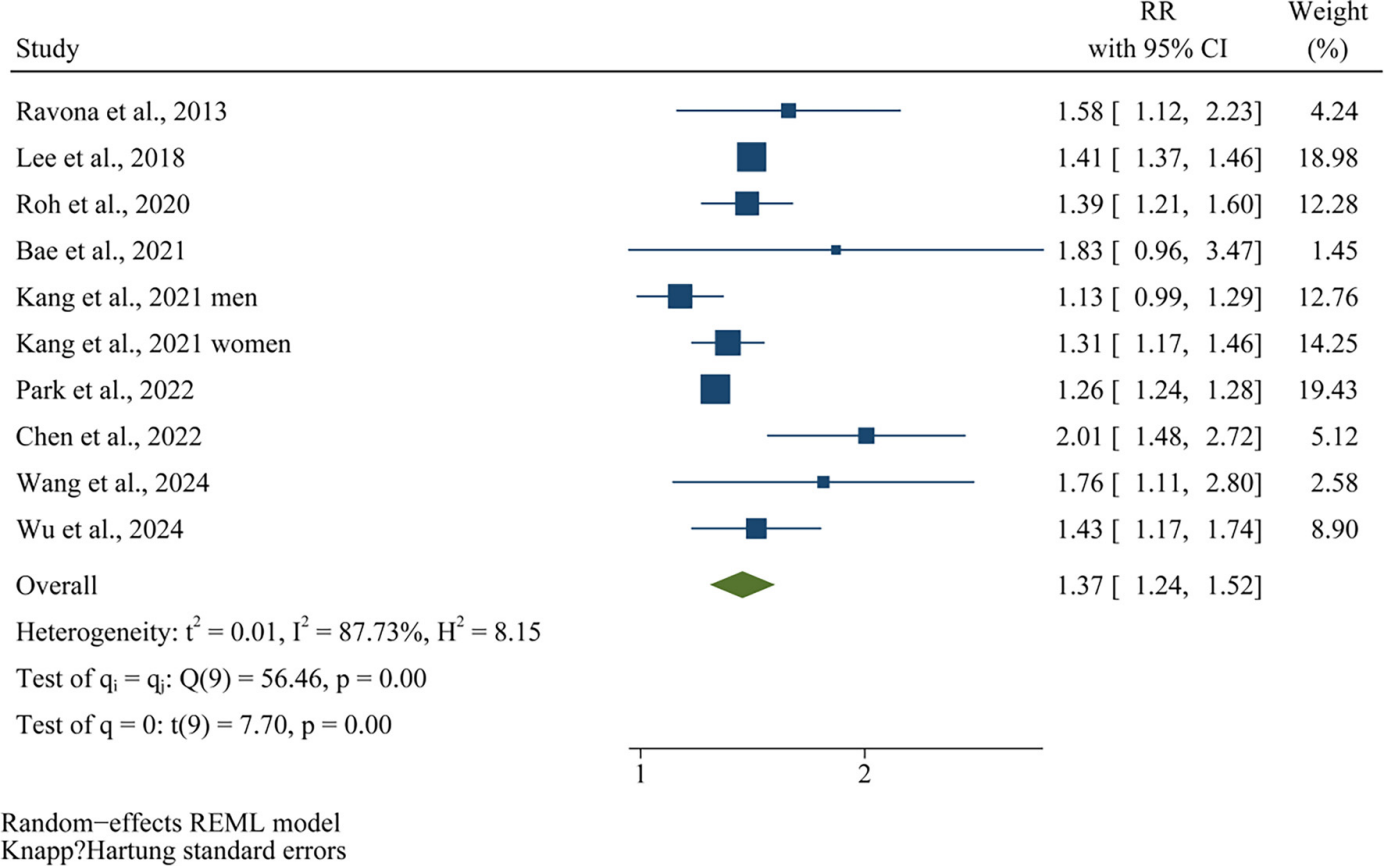
**Forest plots for the sensitivity analysis using the HKSJmethod with REML estimation.** The figure presents a sensitivity analysis of the association between high variability in body weight or BMI and risk of dementia. Using the HKSJ method with REML estimation, the pooled risk ratio was 1.37 (95% CI: 1.24–1.52; *P* < 0.001), with substantial heterogeneity across studies (*I^2^* ═ 87%), confirming the robustness of the main findings. HKSJ: Hartung-Knapp-Sidik-Jonkman; REML: Restricted maximum likelihood; BMI: Body mass index; CI: Confidence interval; RR: Risk ratio; *I*^2^: I-squared (measure of heterogeneity).

Since one of the included studies [21] reported outcomes for men and women separately, these datasets were independently incorporated into the meta-analysis, resulting in a total of 10 datasets available for quantitative analysis. Pooled analysis using a random-effects model revealed that individuals exhibiting the highest variability in BW or BMI had a significantly elevated risk of developing dementia compared to those with the lowest variability (RR ═ 1.36, 95% CI: 1.27–1.46; *P* < 0.001; Figure 2A), with substantial heterogeneity observed across studies (*I^2^* ═ 84%). Furthermore, a sensitivity analysis employing the HKSJ method with restricted maximum likelihood (REML) estimation yielded consistent results (RR ═ 1.37; 95% CI: 1.24–1.52; *P* < 0.001; *I^2^* ═ 87%; Figure S1).

To assess the robustness of the pooled results, a sensitivity analysis was performed by sequentially omitting each included study. The overall association between high variability in BW or BMI and dementia risk remained statistically significant across all iterations, with pooled RRs ranging from 1.34–1.40, all with *P* < 0.001 (Table 3). The *I*^2^ values varied between 58% and 86%, indicating persistent heterogeneity without substantially altering the direction or magnitude of the effect. Notably, the sensitivity analysis limited to studies of good quality (NOS ≥ 7) [17–20, 22–25] demonstrated similar results (RR ═ 1.42; 95% CI: 1.30–1.55; *P* < 0.001; *I^2^* ═ 87%).

**Table 3:** Sensitivity analyses

	**RR for the association between BW or BMI variability and the risk of dementia**			
**Dataset omitted**	**RR [95% CI]**	***P* for effect**	***I* ^2^**	***P* for Cochrane *Q* test**
Ravona et al., 2013	1.35 [1.26, 1.46]	<0.001	85%	<0.001
Lee et al., 2018	1.35 [1.25, 1.47]	<0.001	61%	0.009
Roh et al., 2020	1.36 [1.26, 1.47]	<0.001	86%	<0.001
Bae et al., 2021	1.36 [1.26, 1.46]	<0.001	86%	<0.001
Kang et al., 2021 men	1.40 [1.30, 1.51]	<0.001	85%	<0.001
Kang et al., 2021 women	1.37 [1.27, 1.49]	<0.001	86%	<0.001
Park et al., 2022	1.39 [1.28, 1.51]	<0.001	58%	0.01
Chen et al., 2022	1.34 [1.25, 1.43]	<0.001	83%	<0.001
Wang et al., 2024	1.35 [1.26, 1.46]	<0.001	85%	<0.001
Wu et al., 2024	1.36 [1.26, 1.46]	<0.001	86%	<0.001

RR: Relative risk; BW: Body weight; BMI: Body mass index; CI: Confidence interval.

Additionally, subgroup analyses by exposure type revealed consistent associations for both BW variability (RR ═ 1.45; 95% CI: 1.23–1.70; *I^2^* ═ 76%) and BMI variability (RR ═ 1.34; 95% CI: 1.22–1.48; *I^2^* ═ 62%), with no significant difference between subgroups (*P* ═ 0.43; Figure 2B). Stratification by dementia subtype indicated that higher variability was associated with an increased risk of both AD (RR ═ 1.32; 95% CI: 1.27–1.38; *I^2^* ═ 45%) and VD (RR ═ 1.40; 95% CI: 1.23–1.59; *I^2^* ═ 83%), with no significant subgroup difference (*P* ═ 0.41; Figure 2C).

**Figure 3.**
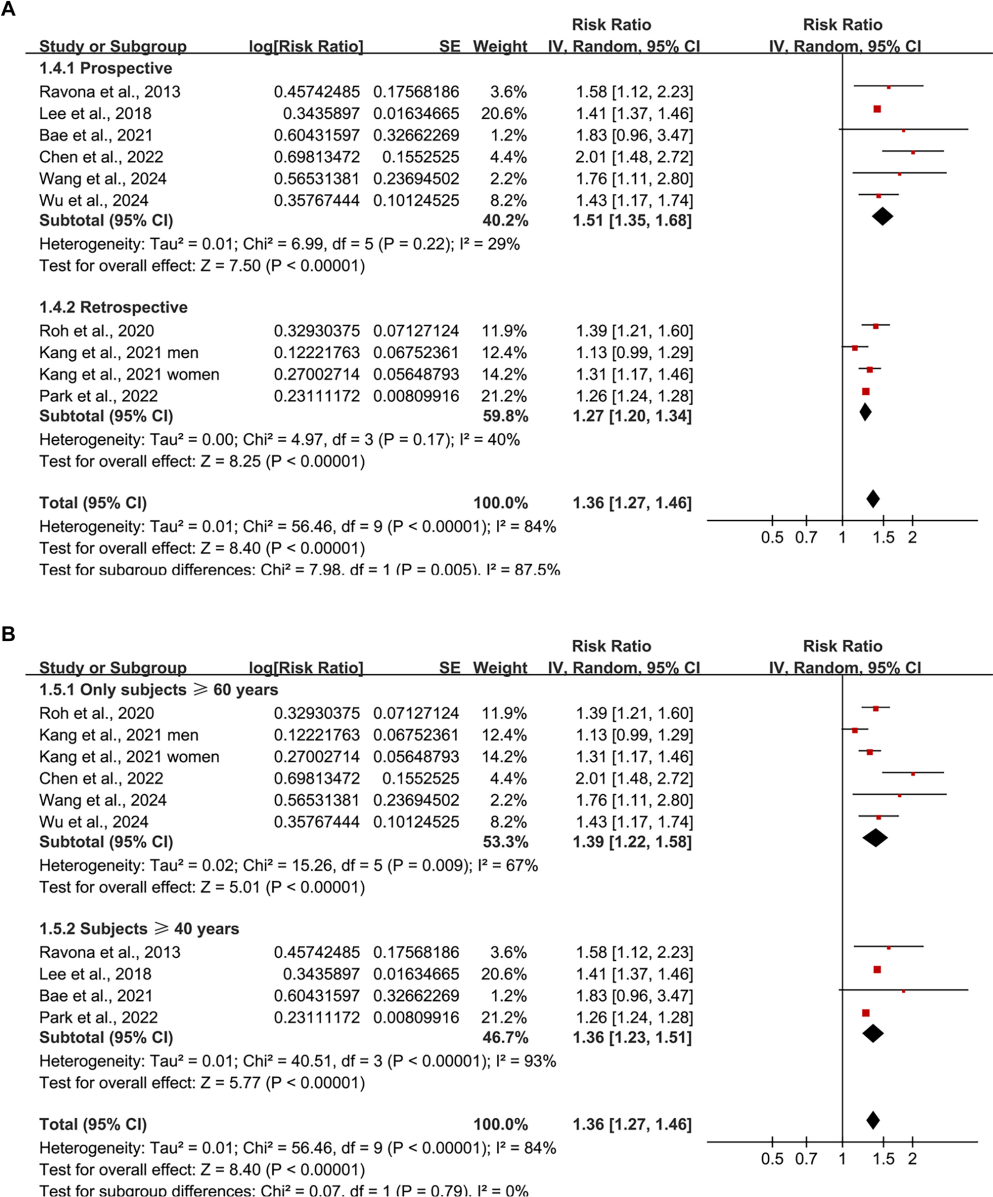
**Subgroup analyses of the association between BW/BMI variability and dementia risk.** (A) Stratified by study design (prospective vs retrospective); (B) Stratified by baseline age of the study population (≥ 60 years vs ≥ 40 years). BW: Body weight; BMI: Body mass index.

**Figure 4.**
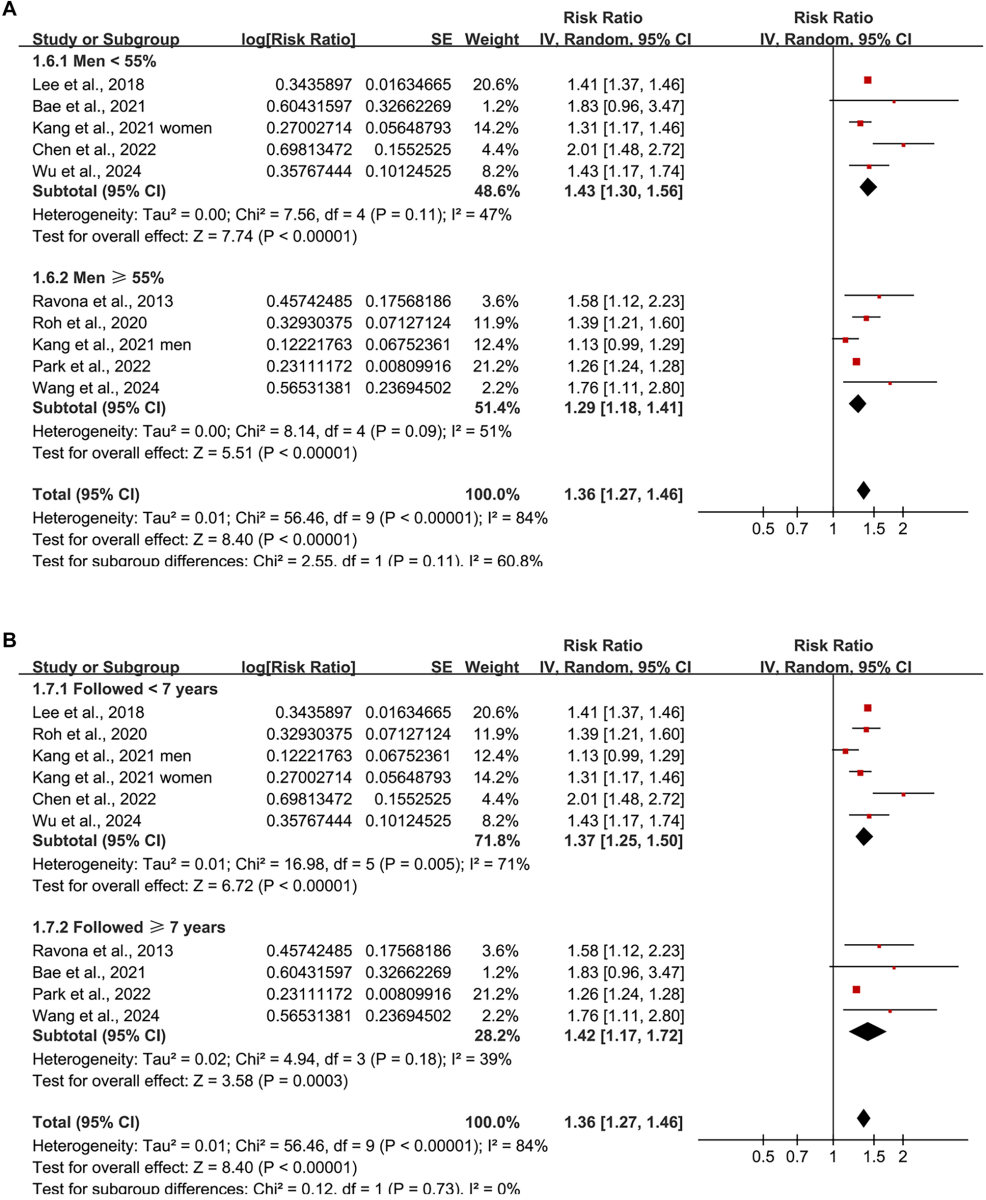
**Subgroup analyses of the association between BW/BMI variability and dementia risk.** (A) Stratified by the proportion of male participants (<55% vs ≥55%); (B) Stratified by follow-up duration (<7 years vs ≥7 years). BW: Body weight; BMI: Body mass index.

**Figure 5.**
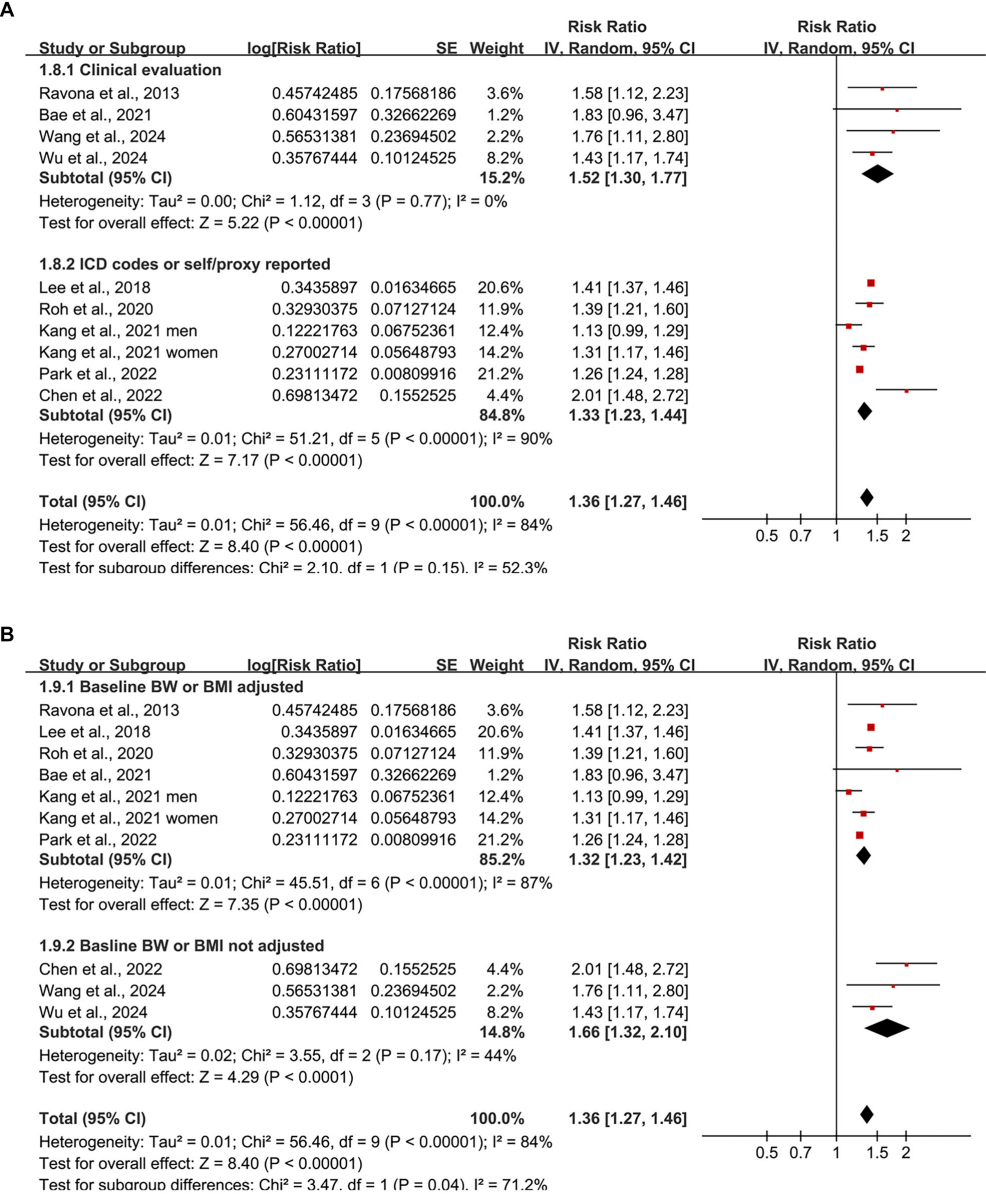
**Subgroup analyses by methodological characteristics.** (A) Stratified by method of dementia diagnosis (clinical assessment vs ICD codes or self/proxy report); (B) Stratified by whether baseline body weight or BMI was adjusted in the analysis. A stronger association was observed in studies that did not adjust for baseline BW/BMI. BW: Body weight; BMI: Body mass index; ICD: International classification of diseases

Further subgroup analyses indicated a stronger association in prospective studies (RR ═ 1.51; 95% CI: 1.35–1.68; *I^2^* ═ 29%) compared to retrospective studies (RR ═ 1.27; 95% CI: 1.20–1.34; *I^2^* ═ 40%), with a significant subgroup difference (*P* ═ 0.005; Figure 3A). Comparable associations were observed in participants aged ≥ 60 years (RR ═ 1.39; 95% CI: 1.22–1.58; *I^2^* ═ 67%) and those aged ≥ 40 years (RR ═ 1.36; 95% CI: 1.23–1.51; *I^2^* ═ 93%; *P* for subgroup difference ═ 0.79; Figure 3B), as well as in studies with < 55% men (RR ═ 1.43; 95% CI: 1.30–1.56) compared to those with ≥ 55% men (RR ═ 1.29; 95% CI: 1.18–1.41; *P* for subgroup difference ═ 0.11; Figure 4A). The association remained consistent across studies with shorter (< 7 years; RR ═ 1.37) and longer (≥ 7 years; RR ═ 1.42) follow-up durations (*P* for subgroup difference ═ 0.73; Figure 4B). Studies utilizing clinical evaluations for dementia diagnosis exhibited a similar association (RR ═ 1.52; 95% CI: 1.30–1.77; *I^2^* ═ 0%) compared to those relying on ICD codes or self/proxy reports (RR ═ 1.33; 95% CI: 1.23–1.44; *I^2^* ═ 90%; *P* for subgroup difference ═ 0.15; Figure 5A). However, the association was significantly stronger in studies that did not adjust for baseline BW or BMI (RR ═ 1.66; 95% CI: 1.32–2.10) compared to those that did (RR ═ 1.32; 95% CI: 1.23–1.42; *P* for subgroup difference ═ 0.04; Figure 5B).

### Publication bias

**Figure 6.**
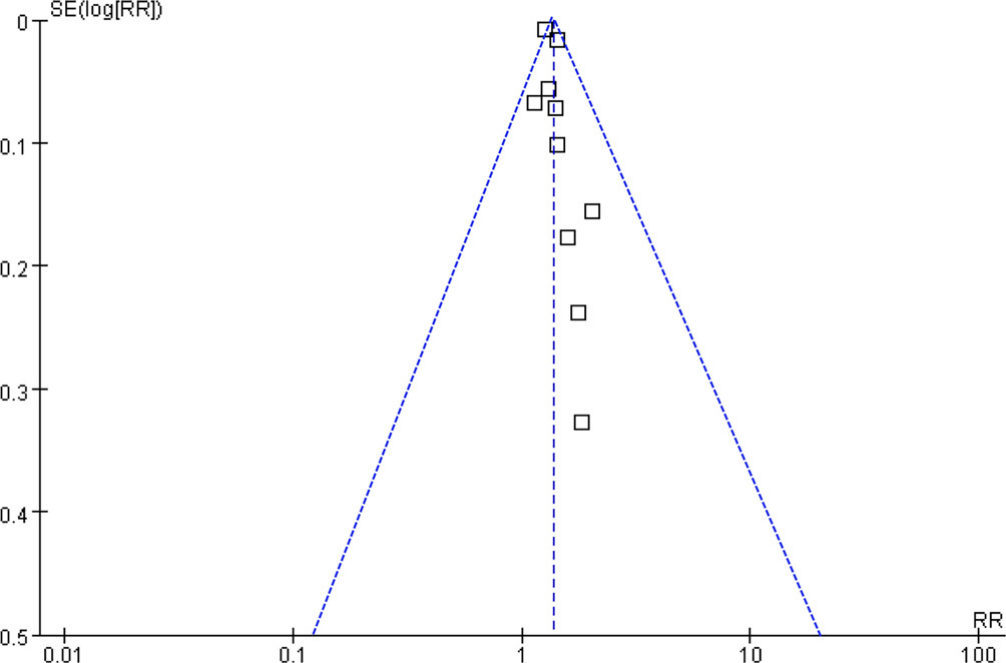
**Funnel plot assessing publication bias.** Funnel plot of studies evaluating the association between BW/BMI variability and dementia risk. Visual symmetry suggests low risk of publication bias, supported by Egger’s regression test (*P* ═ 0.22).

The funnel plots evaluating the relationship between BW or BMI variability and dementia risk are illustrated in Figure 6. A visual inspection of these plots reveals a symmetrical distribution, suggesting a minimal likelihood of publication bias. This finding is further corroborated by Egger’s regression test, which produced a non-significant result (*P* ═ 0.22).

## Discussion

This meta-analysis presents the most current and comprehensive evidence regarding the relationship between intra-individual variability in BW or BMI and the risk of developing incident dementia. Our findings, derived from nine longitudinal cohort studies involving over 4.2 million participants, indicate that increased variability in BW or BMI significantly correlates with a heightened risk of dementia onset. This association was consistent across various dementia subtypes, including AD and VD, and remained robust through numerous subgroup and sensitivity analyses.

The clinical significance of these findings is underscored by the consistency of the observed associations across diverse populations, study designs, and adjustment strategies. Subgroup analyses based on exposure type revealed similar effect sizes for variability in BW and BMI, indicating their interchangeable roles in predicting dementia risk. Notably, even in studies that adjusted for baseline BW or BMI, the association, while attenuated, remained statistically significant. This suggests that the predictive value of weight variability is independent of an individual’s static body composition and reflects more complex underlying physiological or behavioral instability. Research by Liang et al. [32] and Aiken-Morgan et al. [33] supports this interpretation, demonstrating that variability in BMI, rather than baseline BMI alone, is more strongly associated with cognitive decline and the onset of mild cognitive impairment (MCI) in older adults.

Several pathophysiological mechanisms may elucidate the observed relationship between BW/BMI variability and dementia. First, fluctuations in weight may indicate underlying metabolic dysregulation, including impaired glucose tolerance, insulin resistance, and dyslipidemia—factors well-documented as risk contributors to cognitive decline [34, 35]. Second, BW/BMI variability has been correlated with chronic systemic inflammation [36] and altered adipokine signaling [37], both of which can lead to neuroinflammation and dysfunction of the blood-brain barrier. Third, weight fluctuations might signal sarcopenia, malnutrition, or early frailty—conditions associated with neuronal loss and cognitive impairment [38–40]. Notably, Zhou et al. [41] found that greater variability in cardiometabolic and inflammatory markers, including BMI, was independently linked to accelerated cognitive decline in memory and verbal fluency performance over time. Similarly, Kang et al. [42] demonstrated that increased BMI variability correlated with heightened amyloid-β deposition in non-demented individuals, suggesting a potential connection between body composition instability and early AD pathology. Furthermore, the study by Liang et al. (2022) [32] indicated that greater BMI variability predicted faster cognitive decline, even when controlling for mean BMI, highlighting a unique biological pathway through which variability, rather than obesity per se, influences cognition. Our meta-analysis reinforced that the association between BW/BMI variability and dementia was significantly stronger in studies that did not adjust for baseline BW or BMI, although the effect remained statistically significant even in adjusted analyses. This disparity may reflect residual confounding by absolute weight status in the unadjusted subgroup, where individuals with greater variability may also exhibit higher or lower baseline weights, both of which are established risk factors for cognitive decline.

Interpretation of subgroup analyses provides additional insights. The stronger association observed in prospective studies compared to retrospective ones likely reflects improved temporal alignment between exposure and outcome measurement, thereby reducing recall bias and enhancing causal inference [43]. However, it is essential to recognize that early prodromal stages of cognitive decline, such as MCI or subjective cognitive decline, may already be present during the exposure period and influence lifestyle factors, including nutrition and weight regulation [44]. For example, in the extensive study by Lee et al. [18], while dementia diagnoses were made post-variability assessment, early cognitive changes might have remained undetected due to the limitations of administrative databases, complicating causality interpretations. Although associations were observed across various age strata and sex distributions, these characteristics did not significantly alter the effect, suggesting that BW/BMI variability serves as a broadly applicable risk marker. The consistency of results across subgroups defined by follow-up duration and dementia diagnosis methods (e.g., clinical evaluations vs ICD codes or proxy reports) supports the robustness and generalizability of these findings. However, the consistency of associations across AD and VD may also reflect a common endpoint—cognitive decline—rather than a shared etiological mechanism. It is plausible that subtle, preclinical cognitive changes may have already begun to impact daily functioning, appetite, and self-care behaviors, resulting in weight fluctuations independent of dementia subtype [45]. This interpretation underscores the necessity of considering reverse causality in evaluating these findings. Moreover, the observed heterogeneity (*I^2^* ═ 84%) suggests that methodological differences, such as variations in defining variability (e.g., SD vs CV vs VIM), the number and timing of BW/BMI measurements, and the statistical models employed, may contribute to differential estimates and warrant exploration in future individual patient data meta-analyses. Additionally, although three included studies [22, 24, 25] were conducted in Western populations, the majority of the sample was derived from East Asian health-insurance cohorts. Ethnic, lifestyle, and healthcare system differences may influence BW dynamics, access to preventive care, and dementia diagnosis methods. Therefore, caution is advised when generalizing these findings to other regions, particularly where sociocultural and medical practices differ significantly.

This meta-analysis presents several notable strengths. It exclusively includes cohort studies with longitudinal follow-up, thereby minimizing the risk of reverse causality. The large sample size enhances statistical power to detect moderate associations and conduct meaningful subgroup analyses. All included studies employed multivariable-adjusted models, and sensitivity analyses confirmed the stability of results across various study exclusions. Our extensive literature search and rigorous quality assessment using the NOS further bolster the credibility of the findings.

However, several limitations should be acknowledged. First, the heterogeneity among studies was moderate to high, likely attributable to variations in populations, definitions of exposure, and assessments of outcomes. Second, the metrics used to quantify BW/BMI variability were not standardized across studies, which limits comparability and complicates the establishment of clinically actionable thresholds. To our knowledge, an optimal protocol and parameter to accurately reflect the severity of BW variability remain undetermined. Our subgroup analysis based on BW and BMI variability yielded consistent results. Third, although all studies adjusted for key confounders, residual confounding from unmeasured factors (e.g., diet, depression, physical activity, or frailty) cannot be ruled out. Fourth, the reliance on study-level rather than individual-level data precluded exploration of nuanced dose-response relationships or potential non-linear effects. Additionally, the studies did not differentiate between unidirectional changes (i.e., sustained weight loss or gain) and bidirectional fluctuations (i.e., weight cycling), which may have distinct physiological and clinical implications. For instance, weight loss may indicate frailty or malnutrition, while weight gain may signal underlying metabolic dysregulation [46]. Therefore, while our findings suggest that weight instability is associated with an increased risk of dementia, further research is warranted to clarify whether the direction of weight change influences this relationship. In addition, the temporal distribution of weight variability—whether fluctuations occurred rapidly over a short interval or gradually across a longer period—was not consistently reported among studies. This limitation hinders our ability to assess whether short-term instability poses a greater risk than long-term trends. Understanding the timing and clustering of variability may refine surveillance strategies and inform the timing of interventions aimed at stabilizing weight.

Furthermore, although all included studies utilized cohort designs, the possibility of reverse causality cannot be entirely excluded. Dementia has a prolonged preclinical phase, during which subtle cognitive decline may already impact appetite regulation, nutrition, or daily functioning, potentially contributing to BW variability prior to formal diagnosis [47]. Moreover, although temporality was generally ensured by study design, causality cannot be definitively established due to the observational nature of the evidence. Finally, while Egger’s test did not indicate significant publication bias, this finding should be interpreted with caution, given the limited power of asymmetry tests when applied to a small number of studies.

Despite these limitations, our findings carry significant clinical and public health implications. Monitoring BW/BMI variability over time may serve as a simple, non-invasive, and cost-effective tool for identifying individuals at higher risk of dementia, particularly in mid to late life. While guidelines traditionally emphasize achieving a healthy weight, our results suggest that maintaining weight stability may be equally crucial for preserving cognitive health [48]. These findings support the incorporation of longitudinal weight trends into dementia risk models and highlight the potential utility of personalized weight management strategies for cognitive aging prevention. Further studies are warranted to elucidate the importance of maintaining weight stability on cognitive function.

## Conclusion

In conclusion, this meta-analysis demonstrates that greater intra-individual variability in BW or BMI may be independently associated with a higher risk of developing dementia. Given the observational nature of the included studies and the possibility of residual confounding, the overall certainty of the evidence should be considered low to moderate.These findings support the hypothesis that weight instability reflects underlying physiological disturbances that may contribute to neurodegeneration. Future research should aim to clarify causal pathways, define optimal variability thresholds, and evaluate whether interventions targeting weight stability can mitigate dementia risk.

## Supplemental data

**Supplemental File 1 Detailed search strategy for each database**

**PubMed**

(“Body Weight”[Mesh] OR “Body Mass Index”[Mesh] OR “body weight” OR “body mass index” OR BMI) AND (“Variability” OR “variation” OR “fluctuation” OR “oscillation” OR “fluctuate”) AND (“Dementia”[Mesh] OR “Alzheimer Disease”[Mesh] OR dementia OR Alzheimer OR “Alzheimer’s” OR “cognitive decline” OR “cognitive impairment” OR “cognitive dysfunction” OR cognition) AND (“Prospective Studies”[Mesh] OR prospective OR prospectively OR longitudinal OR incident OR incidence OR risk OR followed OR “follow-up” OR cohort)

**Embase**

(‘body weight’/exp OR ‘body mass index’/exp OR ‘body weight’ OR ‘body mass index’ OR BMI) AND (‘variability’ OR ‘variation’ OR ‘fluctuation’ OR ‘oscillation’ OR ‘fluctuate’) AND (‘dementia’/exp OR ‘Alzheimer disease’/exp OR dementia OR Alzheimer OR “Alzheimer’s” OR ‘cognitive decline’ OR ‘cognitive impairment’ OR ‘cognitive dysfunction’ OR cognition) AND (‘prospective study’/exp OR prospective OR prospectively OR longitudinal OR incident OR incidence OR risk OR followed OR ‘follow-up’ OR cohort)

**Web of Science**

TS=(“body weight” OR “body mass index” OR BMI) AND TS=(“variation” OR “variability” OR “fluctuation” OR “oscillation” OR “fluctuate”) AND TS=(“dementia” OR “Alzheimer” OR “Alzheimer’s” OR “cognitive decline” OR “cognitive impairment” OR “cognitive dysfunction” OR “cognition”) AND TS=(“prospective” OR “prospectively” OR “longitudinal” OR “incident” OR “incidence” OR “risk” OR “followed” OR “follow-up” OR “cohort”)

## Data Availability

All data generated or analyzed during this study are included in this published article.
